# Molecular rearrangements of superelectrophiles

**DOI:** 10.3762/bjoc.7.45

**Published:** 2011-03-23

**Authors:** Douglas A Klumpp

**Affiliations:** 1 Department of Chemistry and Biochemistry, Northern Illinois University, DeKalb, Il 60115

**Keywords:** dication, rearrangement, superacid, superelectrophile

## Abstract

Superelectrophiles are multiply charged cationic species (dications, trications, etc.) which are characterized by their reactions with weak nucleophiles. These reactive intermediates may also undergo a wide variety of rearrangement-type reactions. Superelectrophilic rearrangements are often driven by charge–charge repulsive effects, as these densely charged ions react so as to maximize the distances between charge centers. These rearrangements involve reaction steps similar to monocationic rearrangements, such as alkyl group shifts, Wagner–Meerwein shifts, hydride shifts, ring opening reactions, and other skeletal rearrangements. This review will describe these types of superelectrophilic reactions.

## Introduction

The Knorr cyclization is a classical method for preparing quinol-2-ones from β-ketoamides [1]. In 1964, Staskin published a report describing his studies of the Knorr cyclization and noted that the conversion works best with more than 1.0 equiv of Brønsted or Lewis acids [2]. To explain this observation, he suggested a mechanism involving the double protonation of the β-ketoamide to form a dicationic electrophile. A similar mechanism was proposed in which two molecules of Lewis acid were complexed to the β-ketoamide. This manuscript suggested the importance of dicationic intermediates in organic reactions. Other classical conversions, such as the Grewe cyclization [3], clearly involved reactive dicationic intermediates, but until recently there was little or no recognition of these intermediates.

**Scheme 1:**
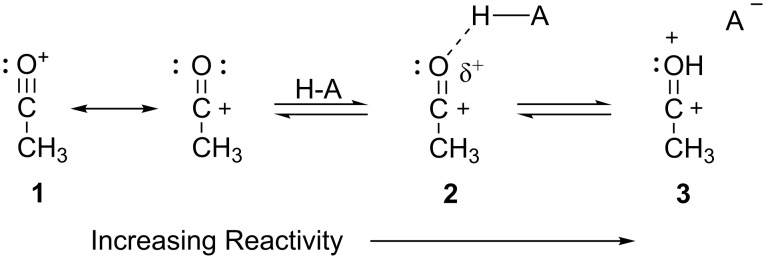
Superelectrophilic activation of the acetyl cation.

During the 1970s, Brouwer and Kiffin reported [4–6] the reactions of branched alkanes with acetyl cation (CH_3_CO^+^) salts in HF·BF_3_. These studies showed that acetyl cation (CH_3_CO^+^) salts were capable of abstracting hydride from the isoalkanes when the reactions were performed in superacdic media. Studies by Olah and coworkers had shown [7] that acetyl cation salts do not react with isoalkanes in aprotic solvents (SO_2_, SO_2_ClF, or CH_2_Cl_2_). To account for the observed increasing electrophilic reactivities, Olah proposed the concept of superelectrophilic activation [7]. It was suggested that the superacidic media interacts with the non-bonding electron pairs of the acetyl cation (**1**), to generate a protosolvated superelectrophile (**2** or **3**, Scheme 1). Protosolvation of the acetyl cation produces an electrophile with increasing dicationic character and consequently superelectrophilic reactivity. Since Olah’s proposal of superelectrophilic activation, the role of dicationic intermediates has become more widely appreciated and it is shown to involve both Brønsted and Lewis acids [8]. Moreover, superelectrophiles have been utilized in many synthetic conversions. While these reactions are often carried out in superacids, less acidic media (CF_3_CO_2_H, H_2_SO_4_, BF_3_·H_2_O, and solid acids) have also been shown to produce superelectrophiles [9–11].

Olah has proposed [8] categories for superelectrophiles, organized according to their structures and the approximate distance between the charge centers (Table 1).

**Table 1:** Representative examples and categories of superelectrophiles.

gitionic superelectrophiles			distonic superelectrophiles
*geminal*	*vicinal*	1,3-dicationic	

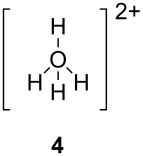	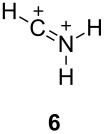	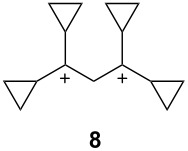	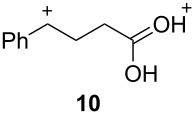
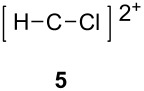	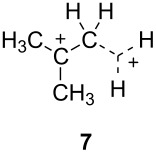	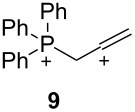	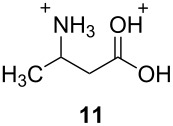

The two basic categories are the *gitionic* and *distonic* superelectrophiles. Gitionic (close) superelectrophiles are characterized by the charge centers being separated by no more than one carbon atom or heteroatom. They are further distinguished by the distance between charges: *Geminal* systems (**4** and **5**) have the charges located around a single atom whilst *vicinal* systems (**6** and **7**) are represented as 1,2-dications. The 1,3-dicationic systems (**8** and **9**) are also considered gitionic superelectrophiles. It is understood that various factors (including charge delocalization) makes such a classification approximate. Distonic (distant) superelectrophiles are characterized by structures having charges separated by 2 or more carbon atoms or heteroatoms (i.e., **10** and **11**). The distonic superelectrophiles are distinguished from other types of onium dications, those in which the onium charge centers are isolated electrophilic sites. In such cases, the onium dications exhibit chemistry that is little different than monocationic electrophiles. Superelectrophiles may also involve hypervalent species, such as protosolvated *tert*-butyl cation (**7**).

Superelectrophiles are characterized by several types of reactions [8]. As very powerful electrophiles, they are best known for their reactions with weak nucleophiles, such as arenes and alkanes. This has led to the development of several new synthetic transformations leading to the functionalization of alkanes. Moreover, superelectrophiles have been used to prepare a wide variety of functionalized arenes. Many types of Friedel–Crafts type reactions have been developed. Among the useful Friedel–Crafts reactions, a large number of cyclizations have been developed, including efficient routes to heterocyclic systems [12]. Several reports have also described superelectrophiles participating in concerted reactions, such as the Nazarov cyclization [13]. Because superelectrophiles are often densely charged species, they are also known for their tendencies to undergo rearrangement and charge migration reactions. These types of conversions will be examined in this review article, including ring opening reactions, carbon–carbon bond shifts, skeletal rearrangements, and charge migrations or hydride shifts. Simple Friedel–Crafts type reactions and cyclizations will not be covered.

## Review

### Ring opening reactions

**Scheme 2:**
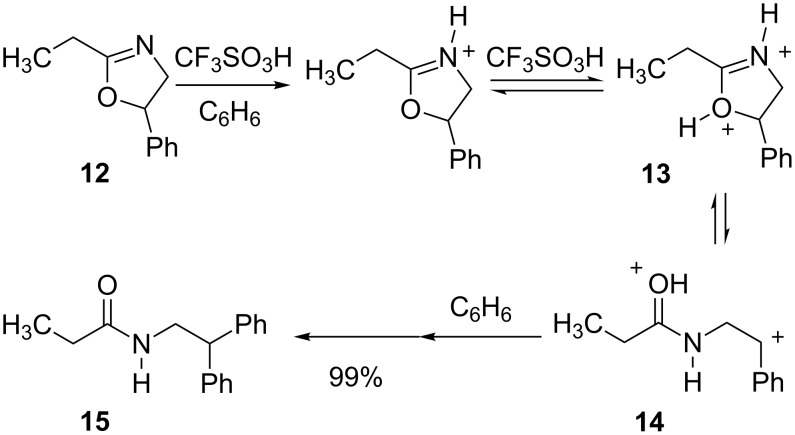
Ring opening of diprotonated 2-oxazolines.

Several types of superelectrophiles are known to undergo ring opening reactions. The ring opening reaction step can be followed by the reaction with a nucleophile. For example, 2-oxazolines were shown [14] to form products with arenes and a mechanism was proposed involving ring opening of the superelectrophile (**13**, Scheme 2).

**Scheme 3:**
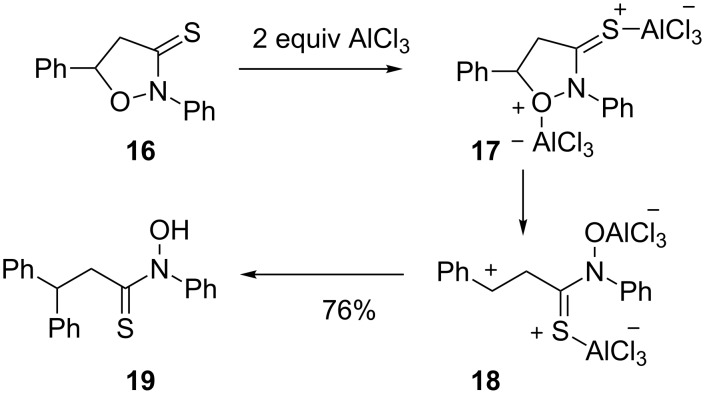
AlCl_3_-promoted ring opening of isoxaolidine **16**.

The ring opening step effectively separates the positive charge centers, as the superelectrophile isomerizes from a 1,3-dication **13** to a 1,5-dication **14**. Subsequent reaction with benzene yields the final product. Using the same chemistry, a chiral 2-oxazoline was shown to give a Friedel–Crafts product in modest diastereoselectivity. A similar reaction was reported [15] in the AlCl_3_-catalyzed reactions of isoxazolidines (Scheme 3). Product conversion required excess amounts of acid, suggesting a mechanism involving superelectrophile **17**. Ring opening to give **18** followed by reaction with benzene afforded **19** in good yield.

**Scheme 4:**
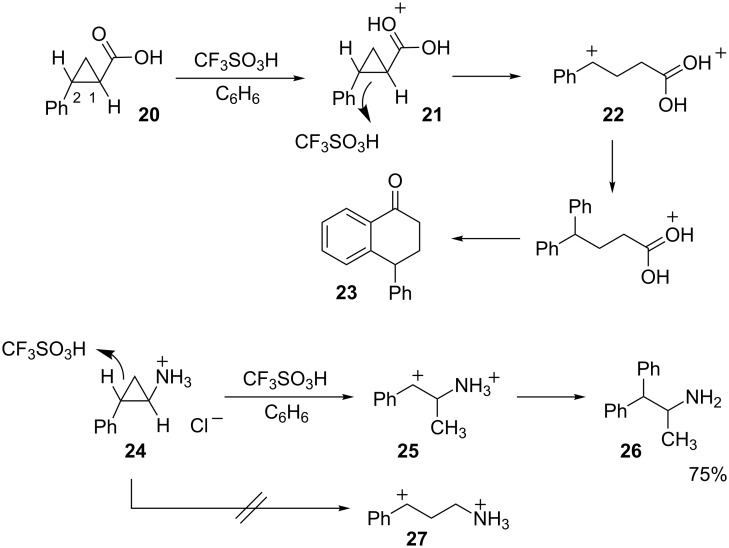
Ring-opening reactions of cyclopropyl derivatives.

In a similar respect, 2-phenylcyclopropanecarboxylic acid (**20**) undergoes diprotonation with ring opening to form the distonic superelectrophile **22** (Scheme 4) [16]. Initial protonation is assumed to occur at the carboxylic acid group to produce **21** followed by protonation of the cyclopropyl ring to give **22**. Protonation at the C1–C2 bond produces a dicationic species with the largest possible charge separation (1,5-dication). Reaction with benzene and cyclization affords the final product **23**. Interestingly, a similar reaction with *trans*-2- phenylcyclopropylamine hydrochloride **24** leads to cleavage of the C2–C3 bond and formation of the 1,3-dication **25** [17]. Protonation of the C1–C2 bond would provide the 1,4-dication **27**, however, this is not observed. It is proposed that the adjacent ammonium group decreases the basicity of the C1–C2 bond in **24**, leading to protonation at the more distant C2–C3 bond. The final product is formed by reaction of benzene at the carbocation site.

**Scheme 5:**
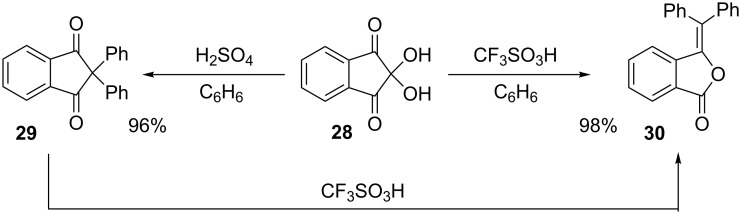
Condensations of ninhydrin (**28**) with benzene.

A number of superelectrophilic ring opening reactions are followed by ring closure steps. For example, ninhydrin (**28**) was shown [18] to give condensation products with arenes in acid-promoted reactions. In H_2_SO_4_, the product **29** is obtained via a simple condensation reaction at the C-2 *gem*-diol group (Scheme 5). When superacidic CF_3_SO_3_H is used, ninhydrin yields 3-(diphenylmethylene)isobenzofuranone (**30**). If product **29** is isolated and then treated with superacid, **30** is obtained as the sole reaction product.

**Scheme 6:**
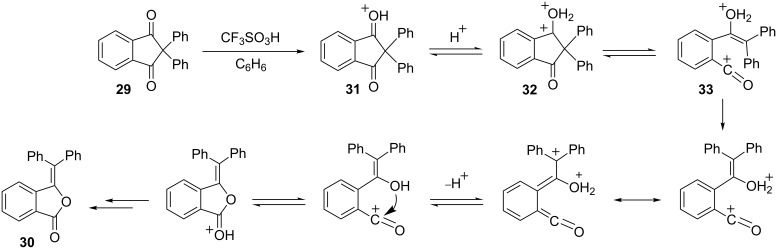
Rearrangement of **29** to **30**.

A mechanism for this conversion is proposed which involves the formation of the *O*,*O*-diprotonated superelectrophile **32** with subsequent ring opening and closing reaction steps (Scheme 6). The mechanism can be understood as a consequence of maximizing charge separation and enabling the charge to have resonance stabilization (i.e., **33**) with two phenyl rings.

**Scheme 7:**
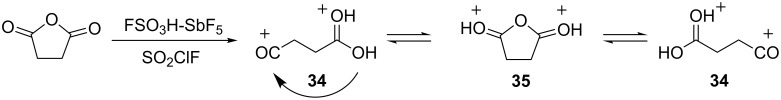
Superacid promoted ring opening of succinic anhydride (**33**).

Succinic anhydride (**33**) reacts in FSO_3_H-SbF_5_-SO_2_ClF solution (Scheme 7) to give the acylium–carboxonium dication **34** which is a stable species at –80 °C [19]. Warming the solution leads to an equilibration between acylium–carboxonium dications and the bis-carboxonium dication.

**Scheme 8:**
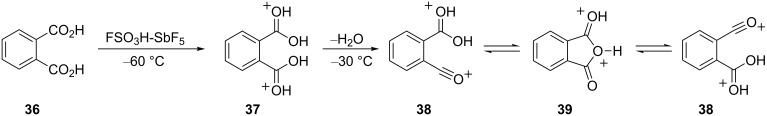
Reaction of phthalic acid (**36**) in FSO_3_H-SbF_5_.

A similar degenerate rearrangement has also been described for glutaric anhydride in superacid. Phthalic acid (**36**) also undergoes a dicationic rearrangement via the anhydride (Scheme 8) [20]. Diprotonated phthalic acid **37** is observed by low temperature ^1^H and ^13^C NMR. When the solution of **37** is warmed, new signals appear which have been assigned to the cleavage product, the acylium–carboxonium dication **38**. NMR evidence suggests the degenerate rearrangement proceeds via the anhydride derivative **39**.

### Carbon migrations and other skeletal rearrangements

**Scheme 9:**
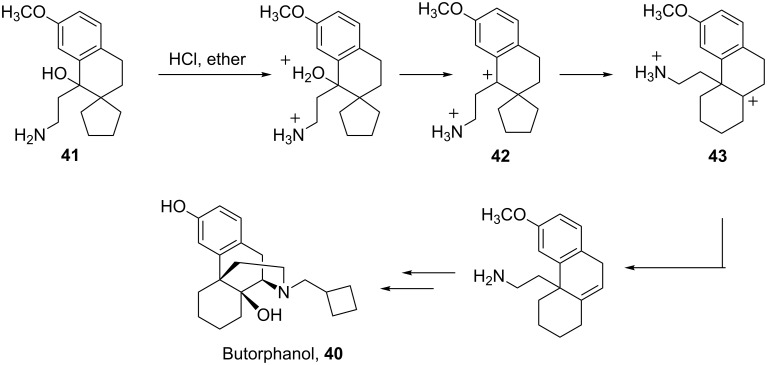
Ring expansion of superelectrophile **42**.

Among the reactions of superelectrophiles, a significant number involve the migration of carbon atoms or heteroatoms. For example, ring expansions reactions have been reported for superelectrophiles. In the synthesis of the analgesic drug butorphanol (**40**) [21], a key step involves the ring expansion of dication **42** to dication **43** (Scheme 9). Interestingly, the ring expansion step moves the carbocationic site away from a benzylic position, but also transforms it from a 1,4-dication to a 1,5-dication. This suggests charge–charge repulsive effects in this system.

**Scheme 10:**
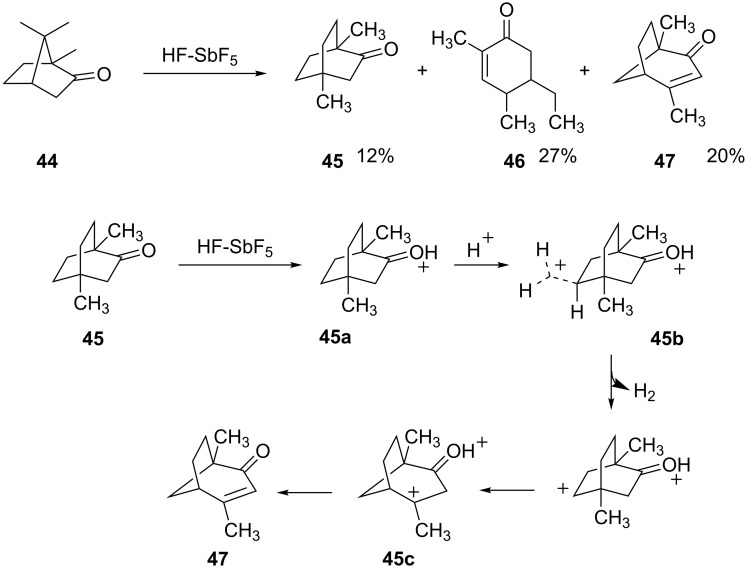
Reaction of camphor (**44**) in superacid.

When camphor (**44**) is reacted with HF-SbF_5_, three products are isolated (Scheme 10) [22]. A mechanism is proposed for conversion of ketone **45** to enone **47**. Initially, the carboxonium ion **45a** is formed by protonation of the carbonyl oxygen. A second protonation occurs in the superacid to produce the carboxonium-carbonium dication **45b** and this species isomerizes to the *tertiary*-carbenium ion by a Wagner–Meerwein shift. Although this isomerization converts a 1,5-dication to a 1,4-dication, the decreasing charge separation is compensated by the formation of a *tertiary*-carbenium ion **45c** which ultimately leads to the stable enone structure **47**.

**Scheme 11:**
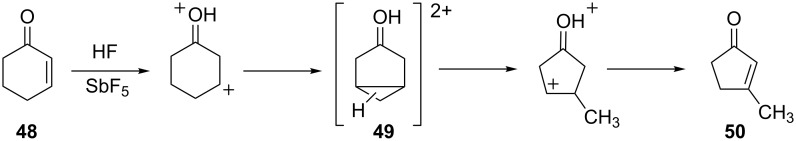
Isomerization of 2-cyclohexen-1-one (**48**).

When 2-cyclohexen-1-one (**47**) is reacted with HF-SbF_5_, a ring contraction occurs to give 3-methyl-2-cyclopenten-1-one (**50**, Scheme 11) [23]. This conversion involves diprotonation of **48** to give a superelectrophile, which undergoes rearrangement via the protonated cyclopropyl derivative **49**. Final reaction steps include a hydride shift and deprotonations to give **50**.

**Scheme 12:**
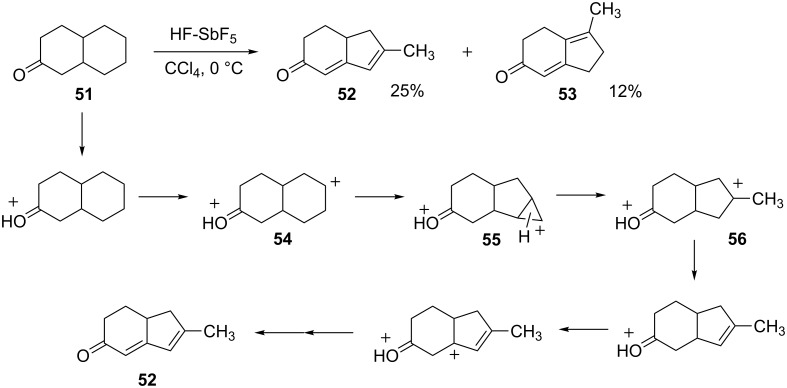
Isomerization of 2-decalone (**51**).

Jacquesy and coworkers [24] have investigated the chemistry of polycyclic ketones in HF-SbF_5_-CCl_4_, a powerful reagent combination for dehydrogenation. For example, 2-decalone (**51**) (primarily *trans*) was reacted with HF-SbF_5_-CCl_4_ at 0 °C to give products **52** and **53** in 25% and 12% yields, respectively (Scheme 12). The proposed mechanism involves protonation and hydride abstraction to give superelectrophile **54**. It is notable that the carbocation forms in the 6- or 7-position on the decalone ring, as this provides maximum separation of the cationic centers. In accord with other carbocation rearrangements, the superelectrophile **54** (a 2° carbocation) isomerizes to **56** (a 3° carbocation). This likely occurs through the ring-fused, protonated cyclopropane **55**. Subsequent deprotonation and hydride abstractions steps give the final product **52**. A similar series of reactions affords the isomeric product **53**.

**Scheme 13:**
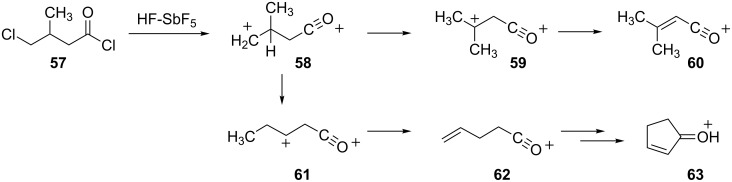
Rearrangement of the acyl-dication **58**.

An interesting rearrangement and cyclization of an acyl dication has been reported [25]. Ionization of the acid chloride **57** in superacid (FSO_3_H-SbF_5_ or HF-SbF_5_) leads to formation of two ions, **60** and **63**, which are observable by NMR spectroscopy (Scheme 13). It was proposed that these ions are formed via superelectrophile **58** by competing hydride and methyl shifts. Following the methyl shift, the acyl cations **61** and **62** are formed. Cyclization then gives the cyclopentenone derivative **63**.

**Scheme 14:**
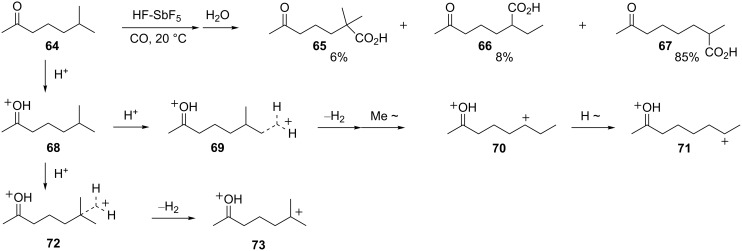
Reaction of dialkylketone **64**.

Carbonylation of superelectrophiles has been shown to give acids and esters in good yields [26]. A rearrangement was described in the carbonylations of dialkyl ketones in HF-SbF_5_ (Scheme 14). Thus, ketone **64** reacts at 20 °C to give **65–67** as products of carbonylation. At this temperature, the rearrangement product **67** is the major product, whilst at −20 °C compound **65** is the major product. The formation of these products can be understood to be the result of two reactions with the superacid: Protonation of the carbonyl group and protolysis of C–H σ-bonds. As a relatively strong base site, the ketone is completely protonated in the superacid to give the carboxonium ion **68**. In the protolysis steps, there is a strong preference to generate the second cationic charge at a site distant from the carboxonium center. Protolysis of the methyl group C–H σ-bonds (i.e., **69**) yields superelectrophile **70** by migration of the methyl group. Protolysis also occurs at the methine position (**72**) which leads directly to superelectrophile **73**. Ions **70** and **73** react with carbon monoxide to give products **66** and **65**, respectively, on aqueous workup. The major product **67** evidently arises from hydride migration in **70** to give dication **71**. This step should be favorable because it increases the distance between charge centers. Although a hydride shift is the most direct route from **70** to **71**, this isomerization may also occur through deprotonation and reprotonation steps. As noted by the authors of this study, protolysis steps with alkanes often leads to β-scission reactions (cleavage of the alkane-based carbocations). This reaction path is not observed with superelectrophiles **70**, **71**, or **73**, because these types of cleavage reactions would generate dicationic species with the cationic charges in closer proximity. Consequently, the carboxonium ion has two interesting effects in this superelectrophilic chemistry. It directs protolysis to the most distant site(s) and it “protects” the alkyl chain from cleavage in the superacid.

**Scheme 15:**
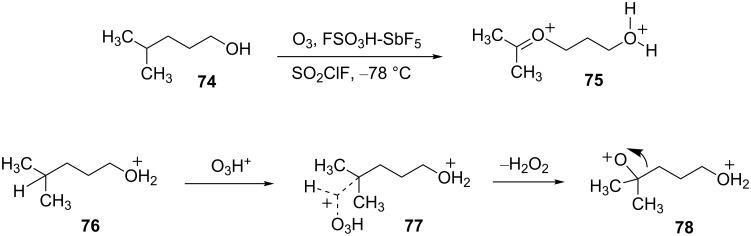
Ozonolysis in superacid.

A series of ozone-based oxidation-rearrangements have been reported by Olah and coworkers some of which involve superelectrophiles [27]. In the presence of Brønsted superacids, ozone is protonated and the resulting ion (O_3_H^+^) is a highly reactive electrophilic species, capable of inserting into C–C and C–H σ-bonds in alkanes or alkyl groups. With another ionizable group present in the substrate, dicationic species can be produced. For example, the pentan-1-ol derivative **74** reacts with ozone in magic acid to yield dication **75** quantitatively (Scheme 15). This conversion involves protonation of the hydroxy group to give the oxonium ion **76** and reaction of O_3_H^+^ at the methine center of **76**. The loss of hydrogen peroxide affords the oxygen-centered cation **78** and subsequent migration of the adjacent group gives dication **75**.

**Scheme 16:**
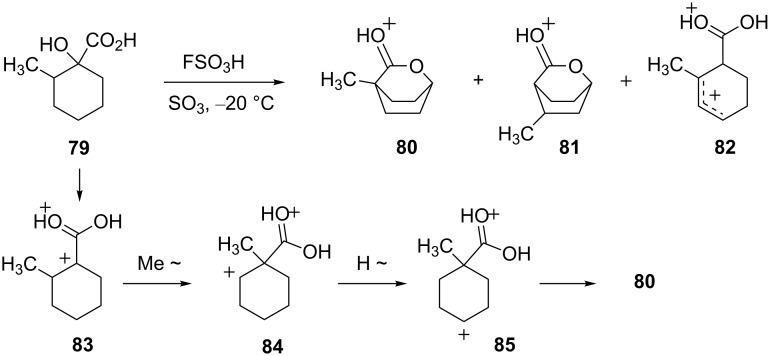
Rearrangement of 1-hydroxy-2-methylcyclohexane carboxylic acid (**79**) in superacid.

Whittaker and Carr have described a series of superacid-promoted reactions to prepare bicyclic lactones [28]. Several of the conversions involve superelectrophilic rearrangements. Ionization of the 1-hydroxy-2-methylcyclohexane carboxylic acid (**79**) in FSO_3_H-SO_3_ at –20 °C leads to a mixture of three ions **80–82** (Scheme 16). For ion **80**, a mechanism was proposed which involves initial formation of superelectrophile **83** followed by methyl group migration to produce dication **84** and successive hydride shifts to give **85** which on ring closure affords the protonated lactone **80**.

**Scheme 17:**
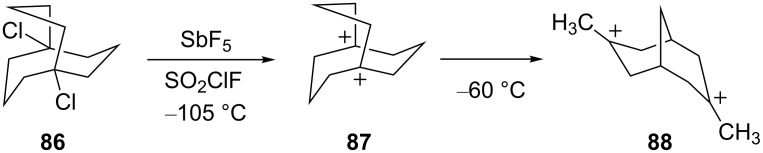
Isomerization of the 1,5-manxyl dication **87**.

A novel isomerization of a bicyclic ring system was described [29] for the 1,5-manxyl dication (**87,** Scheme 17). Ionization of the dichloride **86** in SbF_5_-SO_2_ClF gave the 1,5-manxyl dication (**87**) which was found to be stable at −105 °C. However, upon warming to −60 °C, the 3,7-dimethylbicyclo[3.3.1]nona-3,7-diyl dication (**88**) was formed.

**Scheme 18:**
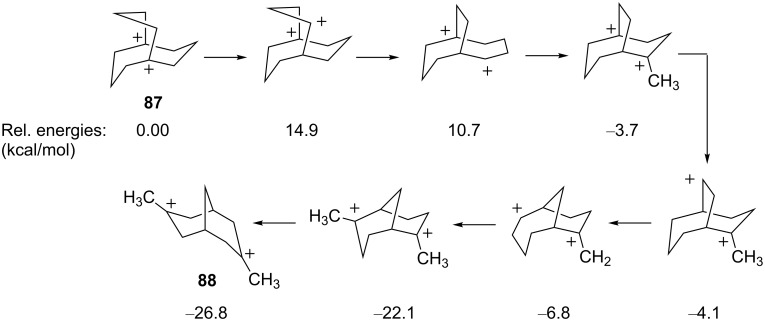
Energetics of isomerization.

This isomerization is thought to occur through a series of hydride shifts, Wagner–Meerwein shifts, ring contractions, and methyl shifts (Scheme 18). Ab initio calculations were performed and revealed that the isomerization lowers the energy of the dication by about 26 kcal/mol. Moreover, isomerization increased the distance between the carbocation sites from 2.80 Å in **87** to 3.58 Å in **88**.

**Scheme 19:**
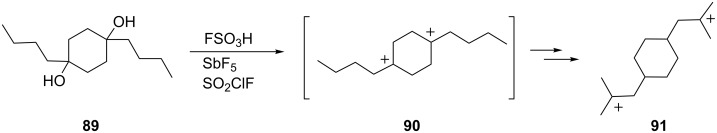
Rearrangement of dication **90**.

Olah and coworkers have described [30] an attempt to generate the 1,4-dication **90** from the disubstituted 1,4-cyclohexanediol (**89**, Scheme 19). The 1,4-dication was not stable and instead isomerized to the 1,8-dication **91**.

**Scheme 20:**
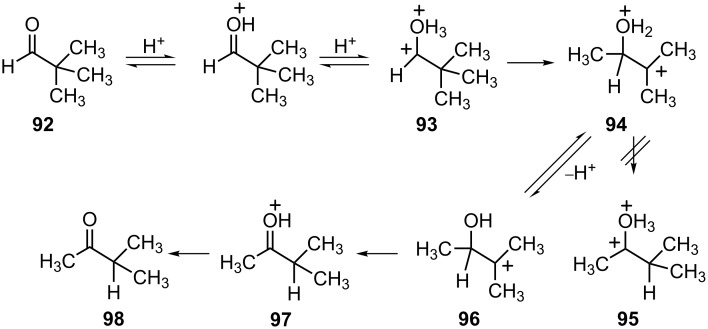
Superacid promoted rearrangement of pivaldehyde (**92**).

Superelectrophilic carboxonium ions are also known to undergo rearrangements by carbon migrations [31]. For example, Olah and Prakash have described the superacid-promoted isomerization of pivaldehyde (**92**) to methyl isopropyl ketone (**98**, Scheme 20).

The rearrangement is best carried out in strong or superacidic media (Table 2). Since the carbonyl group is monoprotonated under less acidic conditions, it suggests the involvement of the *O,O*-diprotonated species **93**. Migration of the methyl group leads to separation of the charge centers and the formation of dication **94**. Theoretical calculations show that hydride shift for the dications (**94** → **95**) is energetically unfavorable, presumably due to the closer proximity of the charges. In the final steps, the monocation **96** undergoes a hydride shift to form the carboxonium ion **97** which leads to ketone **98** on deprotonation. Methyl isopropyl ketone (**98**) is known [32] to be an excellent gasoline oxygenate. Since pivaldehyde can be made from carbon monoxide and isobutane (in superacid), this superelectrophilic rearrangement may have significant commercial value. Other isoalkanes have shown similar chemistry. For example, 3-methylpentane reacts with carbon monoxide to give the isomeric C_7_ ketones.

**Table 2:** Isomerization of pivaldehyde in CF_3_SO_3_H:CF_3_CO_2_H solutions^a^.

*H*_0_	acid system, w/w	pivaldehyde	methyl isopropyl ketone

−10.9	26.9% CF_3_SO_3_H	0%	100%
−9.7	11.4% CF_3_SO_3_H	17%	83%
−9.4	8.0% CF_3_SO_3_H	29%	71%
−8.4	3.1% CF_3_SO_3_H	68%	32%
−7.7	0.9% CF_3_SO_3_H	83%	17%
−2.7	100% CF_3_SO_3_H	100%	0%

^a^Reaction conditions: 2 h, 25 °C, 1:5 pivaldehyde:acid.

**Scheme 21:**
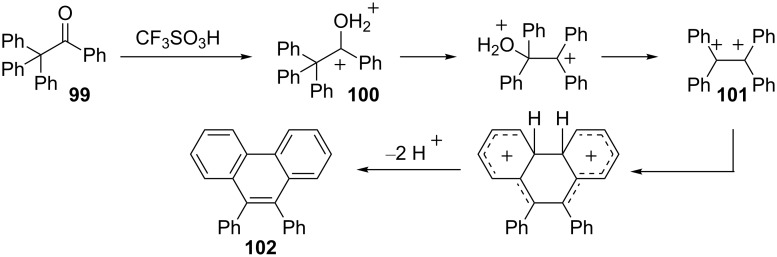
Rearrangement of a superelectrophilic carboxonium ion **100**.

A retro-pinacol rearrangement is triggered by the superelectrophilic carboxonium **100** and subsequent dehydration leads to the efficient formation of the phenanthrene condensation product **102** (Scheme 21) [33]. The key step involves phenyl migration to the carboxonium carbon. This effectively separates and further stabilizes the carbocationic center. Formation of the 1,2-ethylene dication **101** then gives the cyclization product **102**. When dication **101** is generated by other routes, 9,10-diphenylphenanthrene is also formed.

**Scheme 22:**
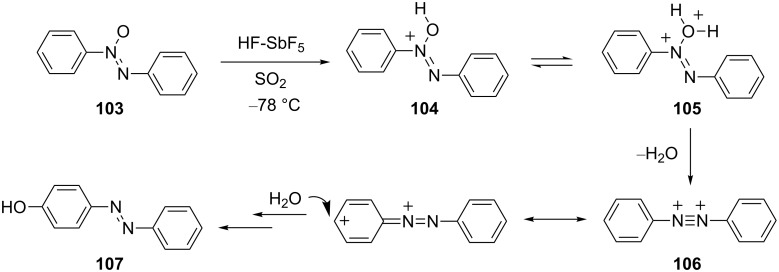
Proposed mechanism for the Wallach rearrangement.

Superelectrophiles are also thought to be involved in some of the classical rearrangements of nitrogen-containing functional groups. For example, Olah and co-workers have studied [34] the Wallach rearrangement and the dicationic intermediates involved were directly observed by low temperature NMR (Scheme 22). Azoxybenzene (**103**) is shown to form the monoprotonated species **104** in FSO_3_H at low temperature, while the dicationic species **105** and **106** are directly observable by NMR in HF-SbF_5_ at low temperature. In the Wallach rearrangement, delocalization of the positive charges is followed by nucleophilic attack by water at a ring carbon during the aqueous workup of the reaction.

**Scheme 23:**
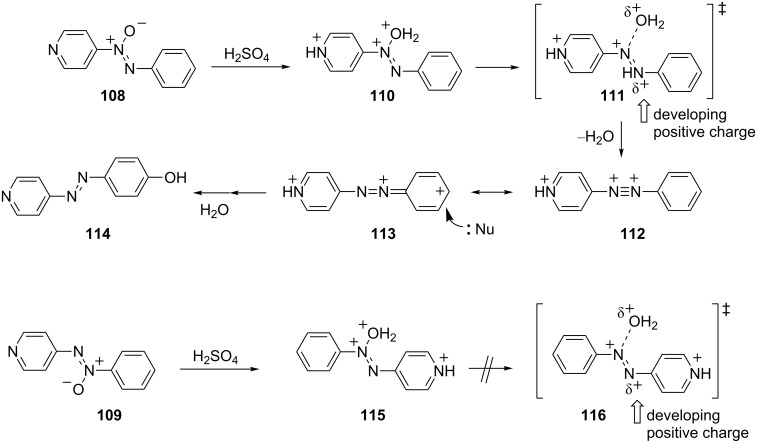
Wallach rearrangement of azoxypyridines **108** and **109**.

An interesting example of the Wallach rearrangement was studied by Buncel and coworkers [35]. In a series of reports, they described the reactions of azoxypyridines in sulfuric acid media. The relative reactivities of the α- and β-isomers **108** and **109** were correlated to stabilization of a developing cationic charge center (Scheme 23). Thus, the α-isomer **108** ionizes in 100% H_2_SO_4_ to give the tricationic species **110** and subsequent nucleophilic attack gives the product **114**. When the β-isomer **104** is reacted under similar conditions, no rearrangement product was obtained. These observations are understood by recognizing that the loss of water from the trications **110** and **115** leads to the development of a positive charge on the adjacent nitrogen atom. In the case of α-isomer **108**, the developing azonium cation may be stabilized by resonance interaction with the phenyl group of **111**. However, with the β-isomer **109** the developing azonium cation is located next to the pyridinium ring **116**. Evidently, structure **116** is destabilized by the unfavorable interactions of cationic charges and the reaction does not occur at a significant rate.

**Scheme 24:**
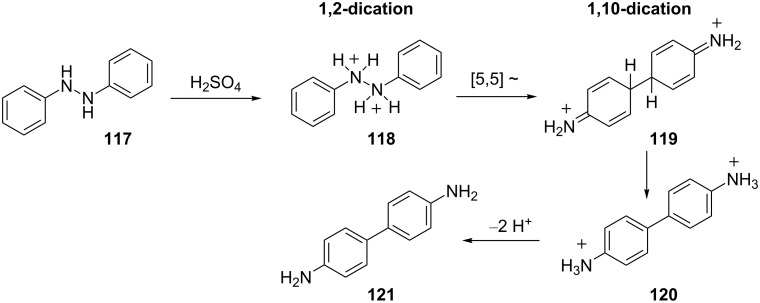
Proposed mechanism of the benzidine rearrangement.

The benzidine rearrangement is another rearrangement that – depending on the reaction conditions – may involve superelectrophiles [36]. In the reaction of 1,2-diphenylhydrazine (**117**), the diprotonated species **118** is formed in strong acid and a 5,5-sigmatropic bond migration occurs (Scheme 24). This step involves the isomerization of the 1,2-dication **118** to the 1,10-dication **119**, a conversion driven to some extent by charge–charge repulsion. The final deprotonation steps give benzidine **121**. Yamabe recently studied the benzidine rearrangement using DFT calculations [37]. The results were in general agreement with the above mechanism: Dication **119** was estimated to be about 9 kcal·mol^−1^ more stable than dication **118** (calculated ions included 12 molecules of water in their structures). Similarly, Olah and coworkers studied this reaction by low temperature NMR and showed clean conversion of hydrazobenzene to the stable ion **119** in FSO_3_H-SO_3_ at –78 °C [34].

**Scheme 25:**
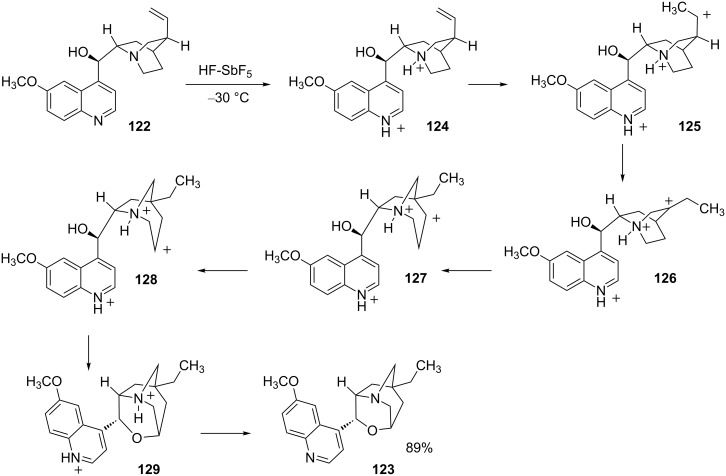
Superacid-promoted reaction of quinine (**122**).

Jacquesy and coworkers have examined the chemistry of natural products in superacids and found several unusual rearrangements of multiply-protonated species. For example, quinine (**122**) gives product **123** in 89% yield from reaction with HF-SbF_5_ at −30 °C (Scheme 25) [38]. The conversion is thought to involve the di- and triprotonated derivatives of quinine **124** and **125**. Hydride and Wagner–Meerwein (WM) shifts lead to formation of trication **127**. Hydride shift gives trication **128**, which undergoes cyclization with the neighboring hydroxy group. This isomerization is somewhat surprising because the 1,4-dicationic system **127** produces a 1,3-dicationic system **128** – generally an energetically unfavorable transformation. This superacid-promoted isomerization of quinine reveals several interesting aspects of the chemistry of structurally complex superelectrophiles. First, protonation of the nitrogen base sites occurs readily and the cationic site may influence the reactivities of adjacent functional groups. This prevents ionization of the hydroxy group and cleavage of the methoxy group, despite being in a superacidic media. Secondly, this example illustrates the challenges in predicting the course of a reaction involving a superelectrophile with a complex structure. There is a very complex interplay of charge–charge repulsions, neighboring group interactions, and other effects.

**Scheme 26:**
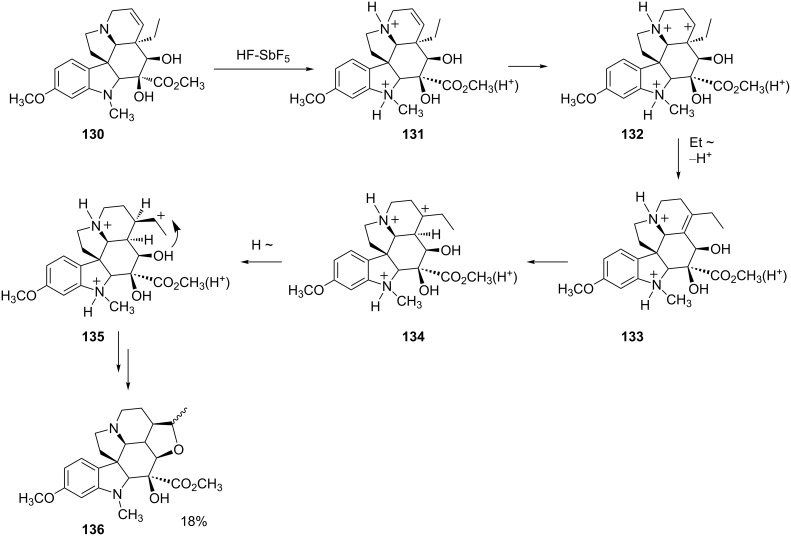
Superacid-promoted reaction of vindoline derivative **130**.

A similar type of rearrangement and cyclization was described [39] for the vindoline derivative **130** in HF-SbF_5_ (Scheme 26). Initial protonation is assumed to occur at the relative strong base sites – the nitrogen atoms and the ester group – to give trication **131**. Further protonation of the double bond leads to carbocation **132**. This intermediate then undergoes an alkyl group shift and deprotonation to give the rearranged alkene **133**. Protonation and charge migration gives ion **135**, which cyclizes to afford **136** as a mixture of diastereomers in 18% yield. Like the rearrangement and cyclization of quinine, this reaction of the vindoline derivative **130** involves a series of structurally complex superelectrophiles. Other superacid-promoted reactions of natural products have been described in recent reviews [40–41].

### Charge migration or hydride shifts

**Scheme 27:**
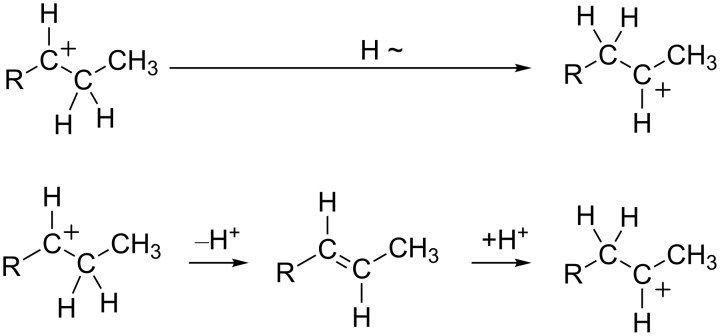
Charge migration by hydride shift and acid–base chemistry.

In the previous section, there were a number of rearrangements that involved both the migration of carbon-centered groups and hydride shifts. The migration of hydride is a common reaction step in carbocation chemistry. Not surprisingly, it also appears to be involved in the chemistry of superelectrophilic systems. There are two means by which charge can migrate in superelectrophiles with the involvement of hydrogen. Charge migration can occur by a direct hydride shift or by deprotonation and protonation steps (Scheme 27). It should be noted that a variety of dicationic superelectrophiles have been shown to exhibit extreme levels of carbon acidity, even undergoing rapid deprotonation in the strongest superacids [42–44]. In general, (di- or tricationic) superelectrophiles tend to favor reactions in which positive charge becomes be more widely dispersed and separated. Reactions are also favored when positive charge can be removed from the structure. Deprotonation can be a means for reducing the overall charge on the superelectrophile. Consequently, the deprotonation–reprotonation may be one of the most common means by which charge migrates in superelectrophiles.

**Scheme 28:**
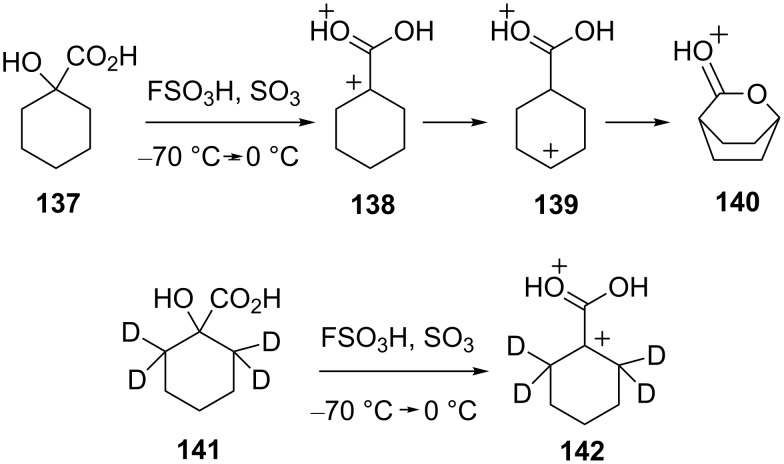
Reactions of 1-hydroxycyclohexanecarboxylic acid (**137**).

Several studies have examined this question using deuterium-labeled superelectrophiles. Reaction of 1-hydroxycyclohexanecarboxylic acid (**137**) in FSO_3_H and SO_3_ at −70 °C, followed by warming to 0 °C, gives a clean conversion to the protonated bicyclic lactone **140** (Scheme 28) [28]. A mechanism is proposed which involves ionization to the superelectrophile **138**, followed by successive hydride shifts to give the charge separated dication **139**. Cyclization then leads to the lactone derivative **140**. In order to further probe this conversion, the deuterium labeled compound **141** was prepared and reacted under similar conditions. Interestingly, a lactone derivative was not formed and only the dicationic species **142** was observed by low temperature NMR. It was proposed that the deuterium atoms slow the initial 1,2-hydride (deuteride) shift and charge migration is inhibited.

**Scheme 29:**
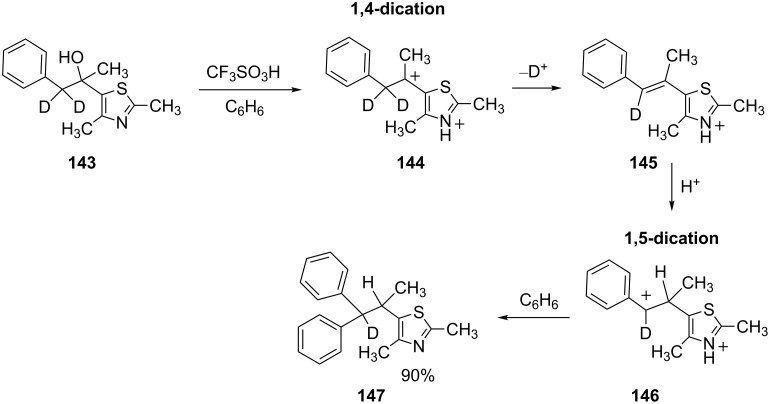
Reaction of alcohol **143** with benzene in superacid.

In another study, the heterocyclic alcohol **143** ionizes in superacid to give the 1,4-dication **144** (Scheme 29) [45]. Further reaction steps lead to the 1,5-dication **146** and ultimately to product **147** in 90% yield. With only one deuterium in the final product, this indicates that charge migration has not occurred by hydride (deuteride) shift, but rather via acid–base chemistry. In this case, the acid–base chemistry may be aided by the formation of a conjugated π-system in **145**.

**Scheme 30:**
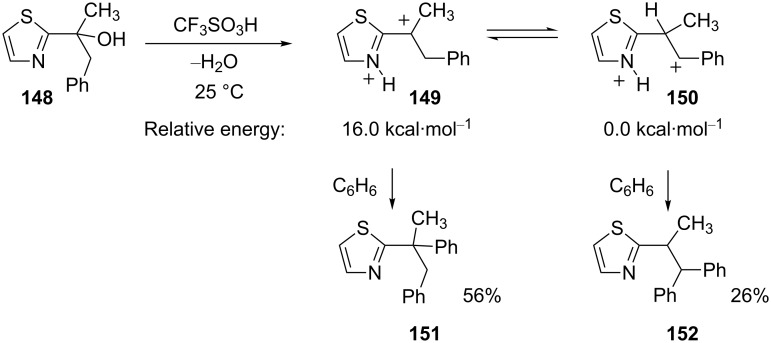
Reaction of alcohol **148** in superacid with benzene.

When cationic charges are in close proximity, it is energetically favorable for the charge centers to be further separated. DFT calculations have performed on several systems and charge separation can result in at least 10–20 kcal·mol^−1^ stabilization. For example, the thiazole derivative **148** was reacted with CF_3_SO_3_H and then benzene to give two products (**151** and **152**, Scheme 30) [42]. When the two precursor superelectrophiles are studied computationally (B3LYP 6-311(d,p) level), the charge separated 1,4-dication **150** is estimated to be about 16 kcal·mol^−1^ more stable than the 1,3-dication **149**. However, since **151** is the major product, this conversion is assumed to be a kinetically controlled reaction. Indeed, compound **152** may be formed exclusively by reacting alcohol **148** in superacid for 1 h, followed by addition of benzene. The initial reaction period enables the superelectrophile to equilibrate and form the more stable charge-separated ion **150**. The addition of benzene then forms **152**.

Another recent study included calculations with the solution-phase model MPW1/6-311G(d)//PCMsp and the solvation was found to narrow the energy gap between a superelectrophile and its charge-separated species (Table 3) [45]. By incorporating the solution-phase into the model, the energy gap between the two ions is decreased by between 3–11 kcal·mol^−1^ compared to gas-phase structures. This result suggests that solvation effects (and almost certainly counter ion effects) are increasingly important in stabilizing superelectrophiles as the ions become more densely charged or the charges are in closer proximity.

**Table 3:** Calculated energies of dications **153** and **154**.

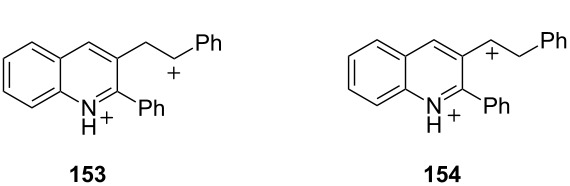		

Level of theory	Relative energy, kcal·mol^−1^	
	**153**	**154**

HF/6-311G (d)	0.0	18.0
B3LYP/6-311G (d)	0.0	14.9
PBE/6-311G (d)	0.0	10.0
MP2/6-311G (d)	0.0	10.3
IPCMsp//MPW1/6-311G (d)	0.0	7.4

**Scheme 31:**
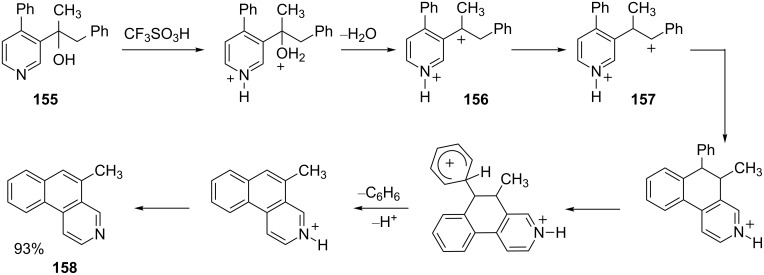
Mechanism of aza-polycyclic aromatic compound formation.

Charge migration and hydride shifts have been involved in several synthetic methods involving superelectrophiles. A useful route to aza-polycyclic aromatic compounds has been developed utilizing charge migration [42,45]. For example, alcohol **155** reacts in superacid to give 5-methylbenzo[*f*]isoquinoline (**158**, Scheme 31) in good yield. This conversion involves formation of the 1,4-dication **156**, which then undergoes charge migration to the 1,5-dication **157**. Intramolecular cyclization and benzene elimination gives the benzo[*f*]isoquinoline system **158**.

**Scheme 32:**
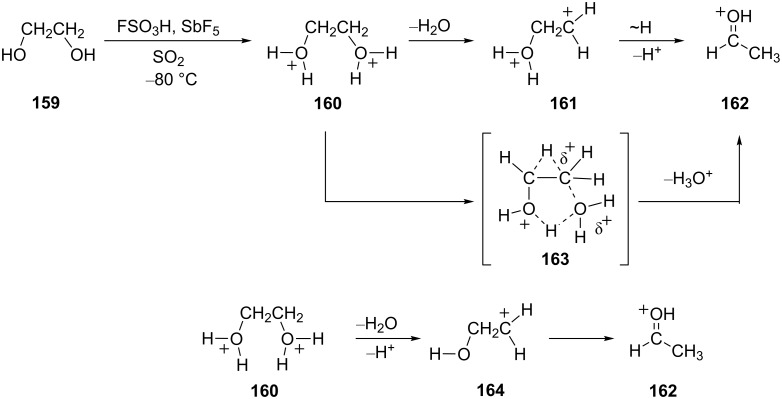
Superacid-promoted reaction of ethylene glycol (**159**).

**Scheme 33:**
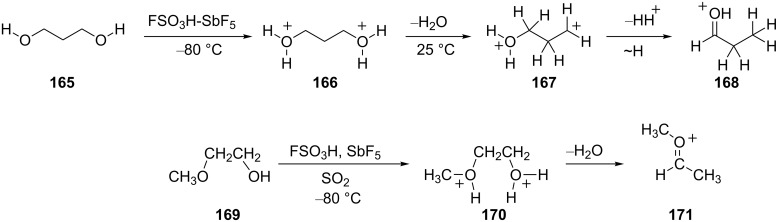
Reactions of 1,3-propanediol (**165**) and 2-methoxyethanol (**169**).

Olah and coworkers have described a series of reactions involving glycols and related substrates in superacids [46]. These substrates are found to give protonated aldehydes and hydride shifts are thought to be involved. In superacidic media, substrates such as ethylene glycol (**159**) are diprotonated and form the bis-oxonium ions, i.e., **160** as a stable species at −80 °C. When the solution is warmed to 25 °C, protonated acetaldehyde (**162**) is formed (Scheme 32). The conversion may occur by one of several routes: by dehydration of **160** with formation of the gitionic superelectrophile **161** and hydride shift/proton loss; by a concerted reaction involving loss of hydronium ion and hydride shift via **163**; dehydration and proton loss with isomerization of the monocationic species **164**. A similar conversion was observed with other substrates such as 1,3-propanediol (**165**) (Scheme 33) and for alkoxy alcohols, i.e., **169**. Both reactions are thought to involve hydride shifts.

**Scheme 34:**
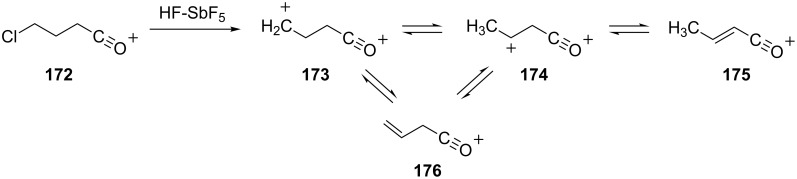
Rearrangement of superelelctrophilic acyl dication **173**.

Reaction of the 4-chlorobutanoyl cation **172** in superacidic HF-SbF_5_ or HSO_3_F-SbF_5_ leads to formation of the 2-butenoyl cation (**175**, Scheme 34) [47]. One of the proposed intermediates in this transformation is the superelectrophilic species **174**, which undergoes deprotonation to give the 2-butenoyl cation **176**. Presumably, **174** is formed by rapid charge migration involving **173**. Further evidence for the superelectrophile **174** is obtained from experiments in which the 2-butenoyl cation **175** is generated in DSO_3_F-SbF_5_. Significant deuterium incorporation is found at the α and γ positions, suggesting equilibria involving **173–176**.

## Conclusion

As a result of their high charge densities, superelectrophiles can exhibit very high reactivities. Superelectrophilic reactivity extends beyond the realm of chemistry with weak nucleophiles. Superelectrophiles may undergo a variety of rearrangement reactions in order to form more stable structures or to lose positive charge. Typically, stabilized structures are characterized by greater separation of cationic charge centers. Superelectrophiles may also undergo structural rearrangements that lead to favorable deprotonation steps. This gives ions with reduced positive charge. Superelectrophiles have been shown to undergo ring opening reactions, alkyl group shifts, Wagner–Meerwein shifts, and hydride shifts. Thus, superelectrophiles tend to rearrange by reaction steps similar to monocationic rearrangements.
